# Systemic restoration of UBA1 ameliorates disease in spinal muscular atrophy

**DOI:** 10.1172/jci.insight.87908

**Published:** 2016-07-21

**Authors:** Rachael A. Powis, Evangelia Karyka, Penelope Boyd, Julien Côme, Ross A. Jones, Yinan Zheng, Eva Szunyogova, Ewout J.N. Groen, Gillian Hunter, Derek Thomson, Thomas M. Wishart, Catherina G. Becker, Simon H. Parson, Cécile Martinat, Mimoun Azzouz, Thomas H. Gillingwater

**Affiliations:** 1 Euan MacDonald Centre for Motor Neurone Disease Research and; 2 Centre for Integrative Physiology, University of Edinburgh, Edinburgh, United Kingdom.; 3 Sheffield Institute for Translational Neuroscience, University of Sheffield, Sheffield, United Kingdom.; 4 INSERM/UEVE UMR861, Institute for Stem cell Therapy and Exploration of Monogenic Diseases (I-Stem), Corbeil-Essonnes, France.; 5 The Institute of Medical Sciences, University of Aberdeen, Aberdeen, United Kingdom.; 6 Department of Life Sciences, Glasgow Caledonian University, Glasgow, United Kingdom.; 7 Centre for Cognitive and Neural Systems,; 8 The Roslin Institute, and; 9 Centre for Neuroregeneration, University of Edinburgh, Edinburgh, United Kingdom.

## Abstract

The autosomal recessive neuromuscular disease spinal muscular atrophy (SMA) is caused by loss of survival motor neuron (SMN) protein. Molecular pathways that are disrupted downstream of SMN therefore represent potentially attractive therapeutic targets for SMA. Here, we demonstrate that therapeutic targeting of ubiquitin pathways disrupted as a consequence of SMN depletion, by increasing levels of one key ubiquitination enzyme (ubiquitin-like modifier activating enzyme 1 [UBA1]), represents a viable approach for treating SMA. Loss of UBA1 was a conserved response across mouse and zebrafish models of SMA as well as in patient induced pluripotent stem cell–derive motor neurons. Restoration of UBA1 was sufficient to rescue motor axon pathology and restore motor performance in SMA zebrafish. Adeno-associated virus serotype 9–UBA1 (AAV9-UBA1) gene therapy delivered systemic increases in UBA1 protein levels that were well tolerated over a prolonged period in healthy control mice. Systemic restoration of UBA1 in SMA mice ameliorated weight loss, increased survival and motor performance, and improved neuromuscular and organ pathology. AAV9-UBA1 therapy was also sufficient to reverse the widespread molecular perturbations in ubiquitin homeostasis that occur during SMA. We conclude that UBA1 represents a safe and effective therapeutic target for the treatment of both neuromuscular and systemic aspects of SMA.

## Abstract 2

Therapeutic targeting of ubiquitin pathways by restoring UBA1 levels is a safe and effective systemic treatment for spinal muscular atrophy.

## Introduction

Proximal spinal muscular atrophy (SMA) is an autosomal recessive neuromuscular disorder that represents a leading genetic cause of infant mortality (1, 2). SMA is caused by low levels of the full-length survival motor neuron (SMN) protein, resulting from mutations or deletions in the *SMN1* gene (3). SMA is primarily characterized by lower motor neuron degeneration and muscle atrophy, although multisystem organ defects are also apparent in severe cases (2, 4). No approved treatment options currently exist for SMA.

As SMA is caused by low levels of SMN, the majority of therapeutic strategies currently under development are aimed at elevating SMN levels in affected cells and tissues (5). However, limitations with SMN-targeted therapies have been highlighted by several animal studies, suggesting that combined therapies that both increase SMN levels and target SMN-independent pathways over the life span will likely be required to develop a fully effective treatment (6, 7).

The ubiquitin proteasome system (UPS) represents one major cellular pathway through which SMN stability is regulated (8, 9). SMN interacts with E3 ubiquitin ligases (10) and deubiquitinating enzymes (11, 12), which can influence SMN levels in in vitro models. Pharmacological inhibition of the proteasome also leads to reduced SMN degradation in vivo and improved neuromuscular pathology in SMA mice (13). In addition, we have previously reported that widespread disruptions in ubiquitin homeostasis are a core feature of SMA pathogenesis (14–16), representing a potential SMN-independent target for therapy development.

Reduced levels of the E1 ubiquitin-like modifier activating enzyme 1 (UBA1) were identified in the neuromuscular system of SMA mice, with suppression of *Uba1* sufficient to recapitulate an SMA-like phenotype in zebrafish (14). Interestingly, mutations in the gene encoding UBA1 (*UBA1*, also known as *UBE1*) cause a very rare, non-SMN-dependent form of SMA in human patients (17–19), indicating a fundamental role for UBA1 in neuromuscular stability and function. Dysregulation of the UPS and Uba1 results in accumulation of downstream UPS target proteins in SMA, including β-catenin (14). Taken together, these findings implicate the UPS and UBA1 as key drivers of SMA pathogenesis, suggesting that they represent a potentially attractive target for therapy development.

In the current study, we investigated whether the loss of UBA1 observed in SMA represents a clinically relevant driver of disease pathogenesis that is amenable to safe therapeutic intervention. We show that suppression of UBA1 is a conserved response to loss of SMN in SMA, including in human SMA patient induced pluripotent stem cell (iPSC) derived motor neurons, with restoration of UBA1 levels in a SMA zebrafish model robustly ameliorating morphological and functional motor defects. AAV9-mediated delivery of UBA1 was safe and well tolerated in control mice, with restoration of UBA1 levels in SMA mice leading to improvements in all neuromuscular and systemic disease parameters investigated and was also sufficient to correct molecular defects in ubiquitin homeostasis. Taken together, these findings represent the first demonstration to our knowledge of a robust, systemic therapy for SMA by targeting ubiquitin pathways.

## Results

### Reduced levels of UBA1 in SMA patient iPSC-derived motor neurons.

**Figure 1:**
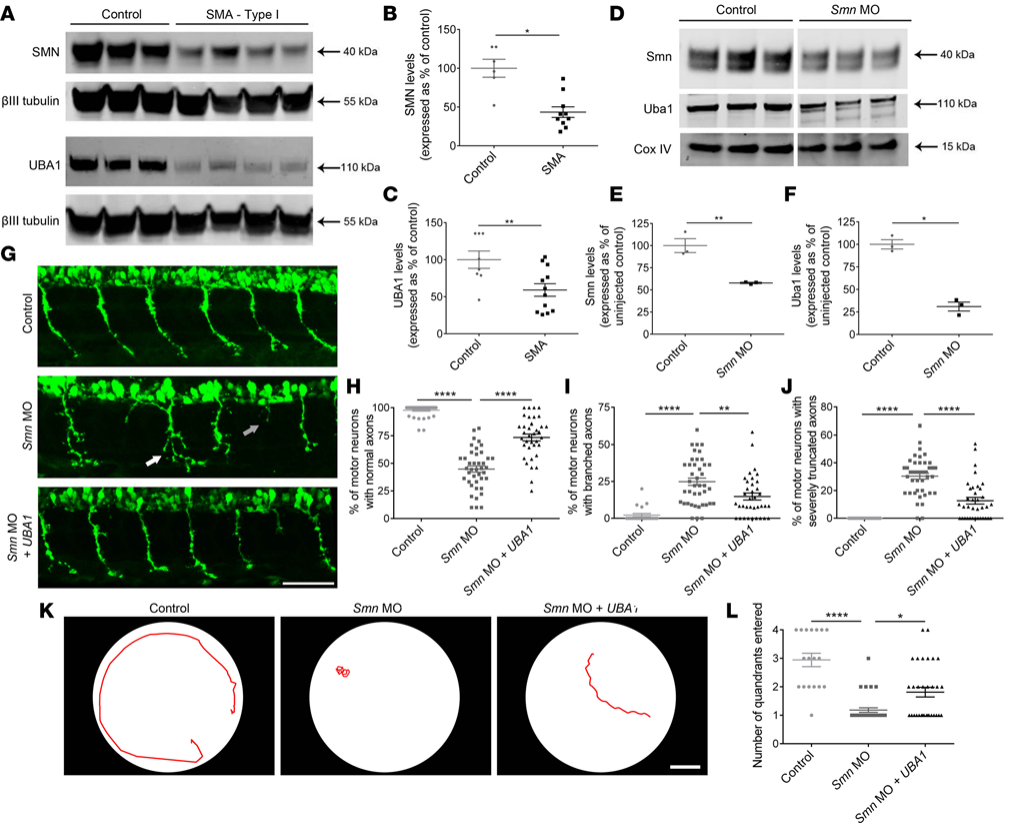
UBA1 loss in patient-derived iPSC motor neurons and SMA zebrafish, with rescue of zebrafish motor pathology following *Uba1* restoration. (**A**–**C**) Significant reduction of survival motor neuron (SMN) and ubiquitin-like modifier activating enzyme 1 (UBA1) protein in type I spinal muscular atrophy (SMA) patient iPSC-derived motor neurons, as quantified by Western blot (independent clones per genotype: control *n =* 9 and SMA *n =* 8; unpaired, 2-tailed Student’s *t* test). (**D**–**F**) Significant reduction in Smn and Uba1 protein levels in zebrafish injected with 6 ng morpholino oligonucleotide (MO) targeted against *Smn* compared to uninjected controls, as quantified Western blot analysis (*n =* 3, batches of 30 fish per lane; unpaired, 2-tailed Student’s *t* test). Lanes were run on the same gel but were noncontiguous. (**G**) Representative micrographs of spinal motor axons from uninjected control zebrafish, zebrafish injected with 4 ng *Smn* MO (white arrow indicates abnormal axon branching, gray arrow indicates severely truncated axons), and zebrafish injected with 4 ng *Smn* MO coinjected 200 ng/μl human *UBA1* mRNA at 30 hours after fertilization (scale bar: 50 μm). (**H**–**J**) Significant improvement in the percentage of normal, branched, and severely truncated motor axons in zebrafish injected with 4 ng *Smn* MO coinjected 200 ng/μl human *UBA1* mRNA (*n =* 20 per treatment group; 1-way ANOVA with Tukey’s post-hoc test). (**K**) Representative tracings of automated swim path analysis of uninjected control zebrafish, zebrafish injected with 4 ng *Smn* MO, and zebrafish injected with 4 ng *Smn* MO coinjected 200 ng/μl human *UBA1* mRNA at 3 days after fertilization (scale bar: 1 cm). (**L**) Significant improvement in the number of quadrants entered during automated swim path analysis of zebrafish injected with 4 ng *Smn* MO coinjected 200 ng/μl human *UBA1* mRNA (control *n =* 18, *Smn* MO *n =* 33, *Smn* MO + UBA1 *n =* 32; Kruskal-Wallis test with Dunn’s post-hoc test). *P ≤ 0.05, **P ≤ 0.01, ****P ≤ 0.001.

Previous studies have reported reduced levels of UBA1 protein in SMA animal models (14), but it remains unknown whether similar changes occur in SMA patient motor neurons. To validate UBA1 as a therapeutic target in human SMA, we measured UBA1 protein levels in induced pluripotent stem cell–derived (iPSC-derived) motor neurons generated from type I SMA patients and controls (20). Quantitative Western blot analysis confirmed a significant reduction in SMN protein levels in SMA patient iPSCs compared with unaffected controls, accompanied by an approximately 40% reduction in UBA1 protein levels (Figure 1, A–C). Taken together with other recent iPSC studies (21), this finding confirms that UBA1 suppression represents a clinically relevant, conserved response to loss of SMN protein across species, highlighting the potential to translate findings from animal models into human patients.

### Restoration of Uba1 rescues motor phenotypes in SMA zebrafish.

It has previously been demonstrated that pharmacologic or genetic suppression of *Uba1* is sufficient to recapitulate the SMA motor phenotype generated by *Smn* knockdown in zebrafish (14). We therefore used the zebrafish SMA model system as an initial in vivo platform to investigate whether experimental restoration of Uba1 has the potential to ameliorate the SMA phenotype. Western blot analysis revealed an approximately 70% reduction in Uba1 protein levels in zebrafish following *Smn* knockdown compared with controls (Figure 1, D–F). Coinjection of full-length human *UBA1* mRNA ameliorated motor axon morphological defects in SMA zebrafish, significantly reducing the incidence of abnormally branched or truncated axons (Figure 1, G–J). In addition, coinjection of *UBA1* mRNA improved the functional motor capacity of SMA zebrafish (Figure 1, K and L). Thus, experimental restoration of Uba1 levels robustly rescued functional and morphological motor defects in a vertebrate model of SMA.

### AAV9-UBA1 delivers a safe, systemic increase in UBA1 levels.

Due to the promising phenotypic improvements observed in our zebrafish model, we next wanted to establish whether a similar phenotypic rescue could be generated in SMA mice using a gene therapy approach. Due to the large size of the *UBA1* gene and limited packaging capacity of self-complementary adeno-associated virus (AAV) (22), vector subcloning was constrained to conventional AAV9. Following generation of AAV9 containing a full-length human *UBA1* open reading frame (AAV9-UBA1; see Methods; Supplemental Figure 1; supplemental material available online with this article; doi:10.1172/jci.insight.87908DS1), we wanted to establish whether we could systemically increase UBA1 levels in vivo and determine whether increased levels of UBA1 are safe and well tolerated.

**Figure 2:**
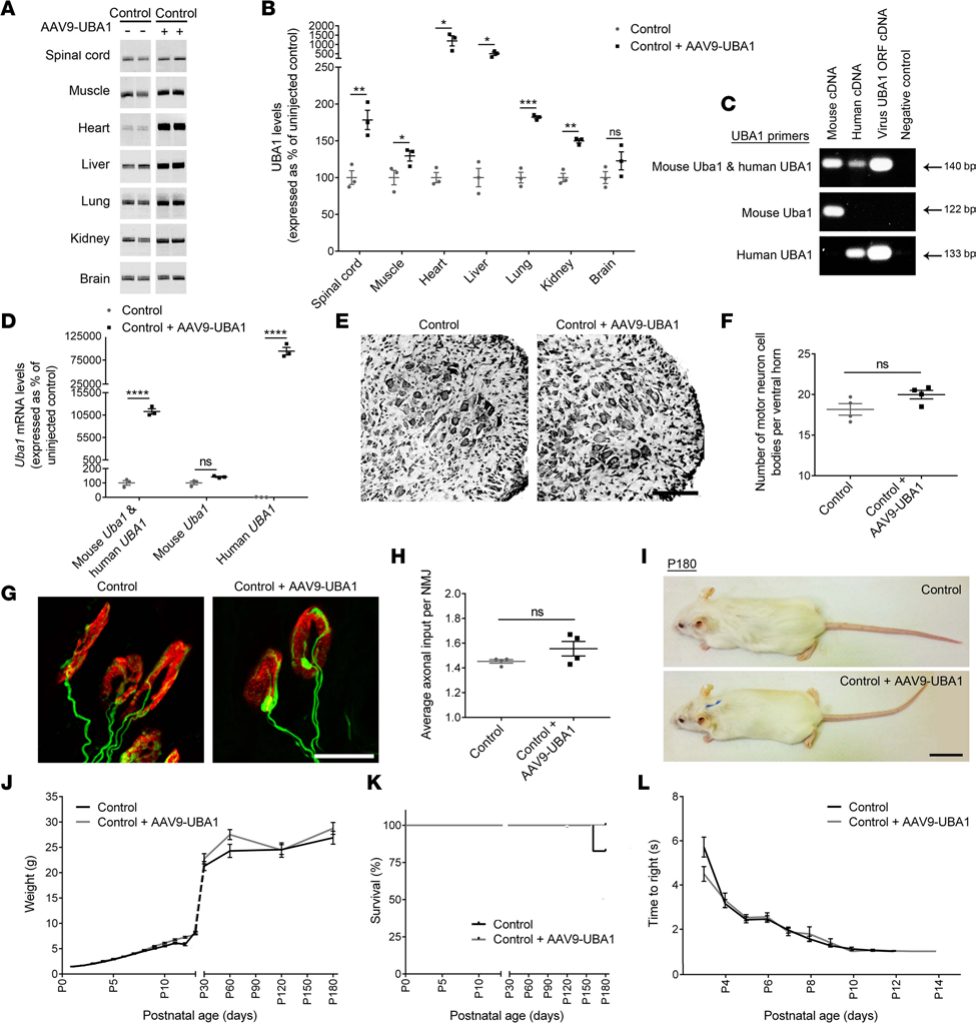
AAV9-UBA1 gene therapy delivers safe systemic increases in UBA1 expression. (**A** and **B**) Adeno-associated virus serotype 9–ubiquitin-like modifier activating enzyme 1 (AAV9-UBA1) delivered at P1 significantly increased UBA1 protein levels in spinal cord, gastrocnemius muscle, heart, liver, lung, and kidney in P7 control mice (*n* = 3 mice per group; unpaired 2-tailed Student’s *t* test). Lanes were run on the same gel but were noncontiguous. (**C**) PCR products following mouse, human, and UBA1 viral cDNA amplification using primers detecting both mouse *Uba1* and human *UBA1* cDNA (top row), unique to mouse *Uba1* cDNA (middle row), and unique to human *UBA1* cDNA (bottom row). (**D**) Significant increase in AAV-expressed human *UBA1* mRNA, but not endogenous mouse *Uba1* mRNA, in the hearts of P7 AAV9-UBA1–treated mice (*n* = 3 mice per group; unpaired, 2-tailed Student’s *t* test). (**E**) Representative Nissl-stained spinal cords (ventral horn) from uninjected control and AAV9-UBA1–treated control mice at P9 (scale bar: 250 μm). (**F**) No significant change in the number of motor neurons in the spinal cords of AAV9-UBA1–treated control P9 mice (*n* = 4 mice per group; unpaired, 2-tailed Student’s *t* test). (**G**) Representative confocal micrographs of neuromuscular junctions (NMJs) in the external oblique from uninjected control and AAV9-UBA1–treated control mice at P9 (axonal inputs in green; postsynaptic endplates in red; scale bar: 25 μm). (**H**) No significant change in axonal inputs at the NMJ in the external oblique muscle of AAV9-UBA1–treated control mice at P9 (*n* = 4 mice per group; unpaired, 2-tailed Student’s *t* test). (**I**) Uninjected control and AAV9-UBA1–treated control mice at P180 (scale bar: 2 cm). (**J**–**L**) No change in AAV9-UBA1–treated controls from P1 to P180 with respect to (**J**) weight (at P180 *n* = 4 mice per group), (**K)** survival (Kaplan-Meier survival analysis), or (**L**) righting reflex test performance (from P1 to P12–P14; unpaired, 2-tailed Student’s *t* test at each time point). ns (not significant) *P* > 0.05*,* *P ≤ 0.05, **P ≤ 0.01, ***P ≤ 0.005, ****P ≤ 0.001. As part of a different comparative analysis, uninjected littermate control data concerning motor neuron cell body counts (**F**) and NMJ axonal input (**H**) is also shown in Figure 5, B and D, respectively. Weight, survival and righting times (**J**–**L**) for uninjected controls are also shown in Figure 4, A–C.

Initial biodistribution studies using Evans blue dye illustrated that intravenous injection via the facial vein led to superior systemic distribution compared with intraperitoneal injection (Supplemental Figure 2); intravenous injection was therefore utilized for subsequent gene therapy delivery. Intravenous injection of AAV9-UBA1 at P1 into control healthy mice led to significant increases in UBA1 protein levels in the spinal cord, muscle, heart, liver, lung, and kidney by P7 (Figure 2, A and B). No change in UBA1 protein levels was observed in the brain by P7. This finding was consistent with temporal expression profiles obtained from a parallel biodistribution analysis of AAV9-mediated expression in mice, showing that CNS expression was slower to appear than that in other tissues and organs (Supplemental Figure 3, A–E). qRT-PCR analysis using primers designed to specifically detect human or mouse *UBA1* revealed that the UBA1 increase seen in tissue from AAV9-UBA1–injected mice was exclusively attributable to virally delivered human *UBA1* cDNA expression, as mouse *Uba1* mRNA levels remained unchanged (Figure 2, C and D).

Neuropathological analyses of AAV9-UBA1–treated control mice at P9 showed no detectable changes in spinal motor neuron number or morphology (Figure 2, E and F), neuromuscular junction (NMJ) integrity (Figure 2, G and H, and Supplemental Figure 4, A and C), or muscle fiber diameter (Supplemental Figure 4, B and D). In a longer-term safety study, the appearance (Figure 2I), weight (Figure 2J), survival (Figure 2K), and motor performance (Figure 2L) of AAV9-UBA1 mice remained indistinguishable from uninjected control mice throughout (endpoint of P180). To investigate whether any underlying, systemic physiological alterations were induced following AAV9-UBA1 injection, hematology analysis and serum biochemistry screens were conducted. No significant alterations in any of the 19 hematology parameters (Supplemental Table 1) or the 11 serum biochemistry parameters (Supplemental Table 2) were noted in AAV9-UBA1–treated mice. Together, these data illustrate that a robust systemic increase in UBA1 proteins is achievable using AAV9 gene therapy, with the high resulting levels of UBA1 being well tolerated, with no obvious adverse effects.

### Systemic restoration of UBA1 protein levels in SMA mice using AAV9-UBA1.

**Figure 3:**
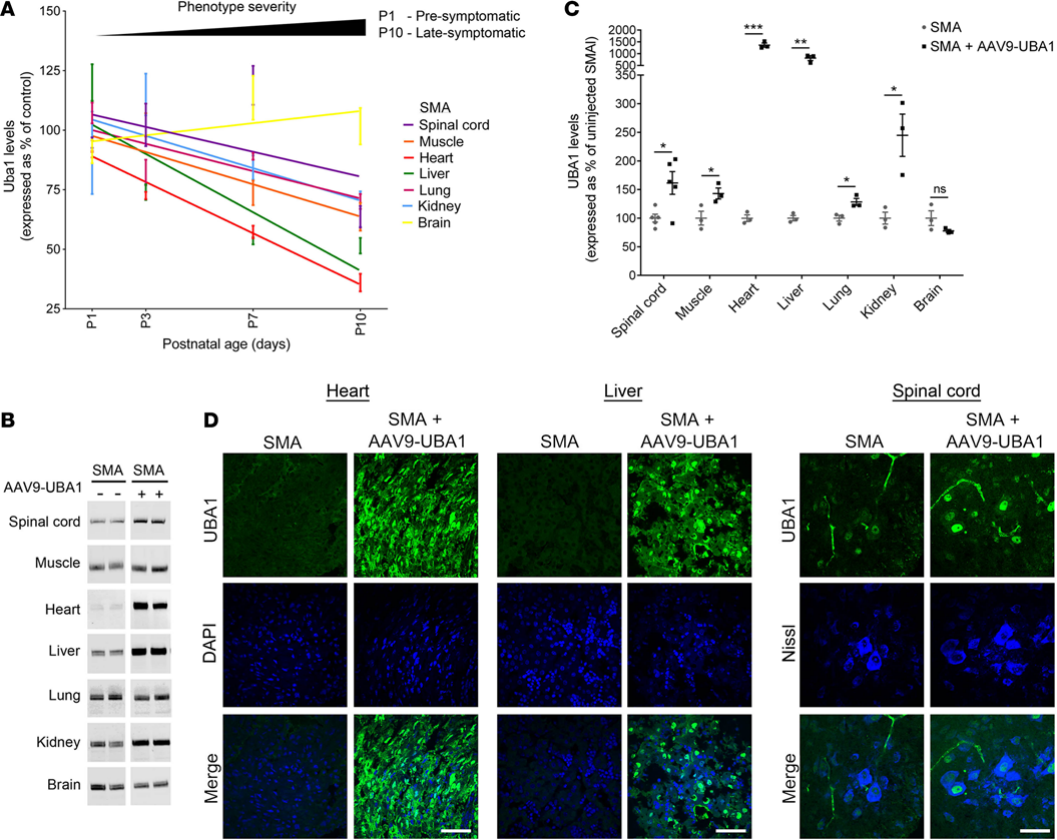
Systemic reductions in Uba1 protein in SMA mice can be restored with AAV9-UBA1 gene therapy. (**A**) Quantification of ubiquitin-like modifier activating enzyme 1 (Uba1) levels in the spinal cord, gastrocnemius muscle, heart, liver, lung, kidney, and brain of spinal muscular atrophy (SMA) mice at P1, P3, P7, and P11 by western blot analysis, expressed as a percentage of control values (n = 3 mice for each genotype at each time point). (**B** and **C**) Intravenous adeno-associated virus serotype 9–UBA1 (AAV9-UBA1) gene therapy at P1 leads to a significant increase in UBA1 protein levels in the spinal cord, gastrocnemius muscle, heart, liver, lung, and kidney (but not whole brain) in P7 SMA mice, as quantified by Western blot (*n* = 3 mice per treatment group, except for spinal cord, for which *n* = 5; unpaired 2-tailed Student’s *t* test). Lanes were run on the same gel but were noncontiguous. (**D**) Representative confocal micrographs showing increased UBA1 levels (green) in the heart, liver, and motor neurons in the spinal cord ventral horn of P7 AAV9-UBA1–treated mice compared to uninjected SMA mice. Hearts and livers were colabeled with DAPI and the spinal cord fluorescent Nissl stain (blue) (scale bar: 50 μm). ns (not significant) *P >* 0.05*,* *P ≤ 0.05, **P ≤ 0.01, ***P ≤ 0.005.

We have previously characterized UBA1 perturbations in the neuromuscular system of SMA mouse models (14). However, considering the growing body of evidence suggesting involvement of other organ systems in SMA pathology (4), we wanted to establish what other organs and tissues also showed UBA1 depletion in “Taiwanese” SMA mice in order to determine critical target sites for delivering AAV9-UBA1. Western blot analysis revealed that all nonneuronal organs examined had perturbations in UBA1 levels, with the heart being particularly affected, followed by the liver, skeletal muscle, kidney, lung, and spinal cord (Figure 3A). Reduced UBA1 levels in organs correlated with disease progression, with changes occurring during presymptomatic/early symptomatic stages of disease (Figure 3A) suggesting that early, systemic delivery of AAV9-UBA1 would be required in SMA mice.

Similar to previous experiments in control mice, intravenous injection of AAV9-UBA1 into “Taiwanese” SMA mice at P1 led to a significant increase in UBA1 protein levels in the spinal cord, muscle, heart, liver, lung, and kidney of control mice by P7 (Figure 3, B and C). Immunohistochemical analysis confirmed increased UBA1 protein levels in the heart, liver, and spinal cord motor neurons of SMA mice treated with AAV9-UBA1, with a predominantly nuclear localization consistent with the presence of a nuclear localization signal in full-length human UBA1 (23) (Figure 3D).

### AAV9-UBA1 improves weight, survival, and motor performance of SMA mice.

**Figure 4:**
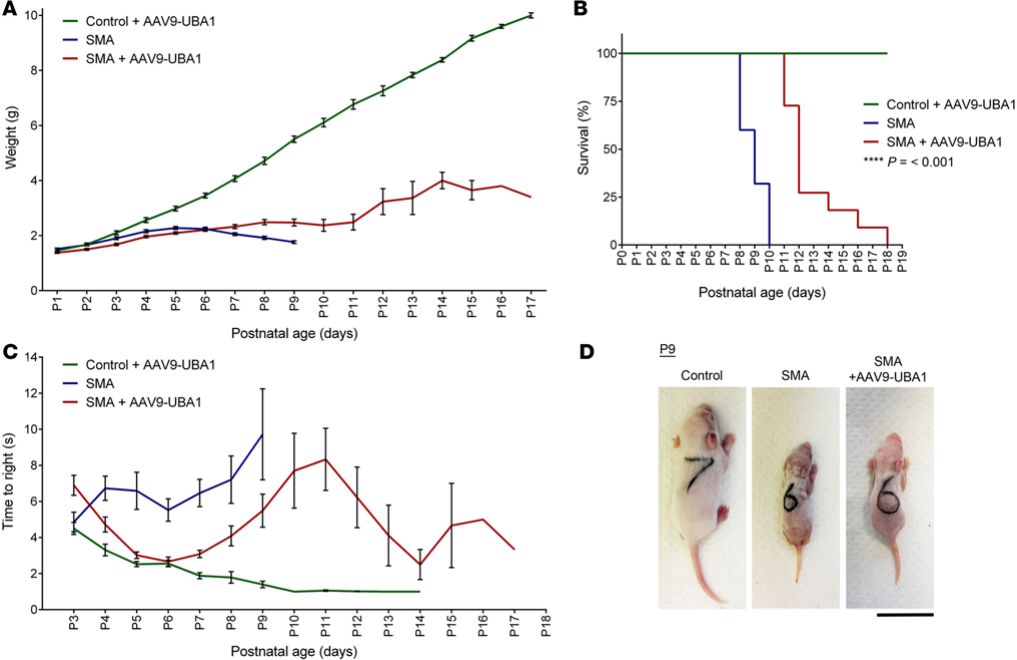
Improved body weight, survival, and motor performance of SMA mice treated with AAV9-UBA1. (**A**) Average weights of adeno-associated virus serotype 9–ubiquitin-like modifier activating enzyme 1–treated (AAV9-UBA1–treated) control mice (*n* = 28 at P3), uninjected spinal muscular atrophy (SMA) mice (*n* = 25 at P3), and AAV9-UBA1–treated SMA mice (*n* = 19 at P3). (**B**) Survival analysis of AAV9-UBA1–treated control mice (*n* = 19), uninjected SMA mice (*n* = 25), and AAV9-UBA1–treated SMA mice (*n* = 11) (Kaplan-Meier survival analysis). (**C**) Average righting reflex times of AAV9-UBA1–treated control mice (*n* = 28 at P3), uninjected SMA mice (*n* = 25 at P3), and AAV9-UBA1–treated SMA mice (*n* = 19 at P3). (**D**) Representative photographs of a AAV9-UBA1–treated control mouse, uninjected SMA mouse, and a AAV9-UBA1–treated SMA mouse at P9 (scale bar: 2 cm). ****P ≤ 0.001. As part of a different comparative analysis, weight, survival and righting times (**A**–**C**) for uninjected controls are also shown in Figure 2, J–L.

To establish whether delivery of AAV9-UBA1 could improve the phenotype of SMA mice, the weight, survival, and motor performance of AAV9-UBA1–treated SMA mice were monitored following intravenous injection at P1. At late-symptomatic ages, the weight of AAV9-UBA1 SMA mice was improved compared with uninjected SMA mice. For example, at P8 AAV9-UBA1–treated mice weighed 2.5 ± 0.1 g (*n* = 15) compared with uninjected SMA mice at 1.9 ± 0.06 g (*n* = 15) (*P* ≤ 0.001) (Figure 4A). Survival of SMA mice was also improved following AAV9-UBA1 delivery, with treated mice displaying a median survival age of P12 (*n* = 11) compared with P9 for uninjected SMA mice (*n* = 25) (*P* ≤ 0.001) (Figure 4B). Righting reflex times (a robust measure of neuromuscular function in neonatal mice) were strikingly improved to near control levels from P4 to P7 in SMA mice treated with AAV9-UBA1, with those mice living to P17 still able to right themselves (Figure 4C). At late-symptomatic stages of disease, AAV9-UBA1–treated SMA mice had a healthier overall appearance compared with untreated SMA animals (Figure 4D).

### AAV9-UBA1 rescues neuromuscular and systemic pathology in SMA mice.

**Figure 5:**
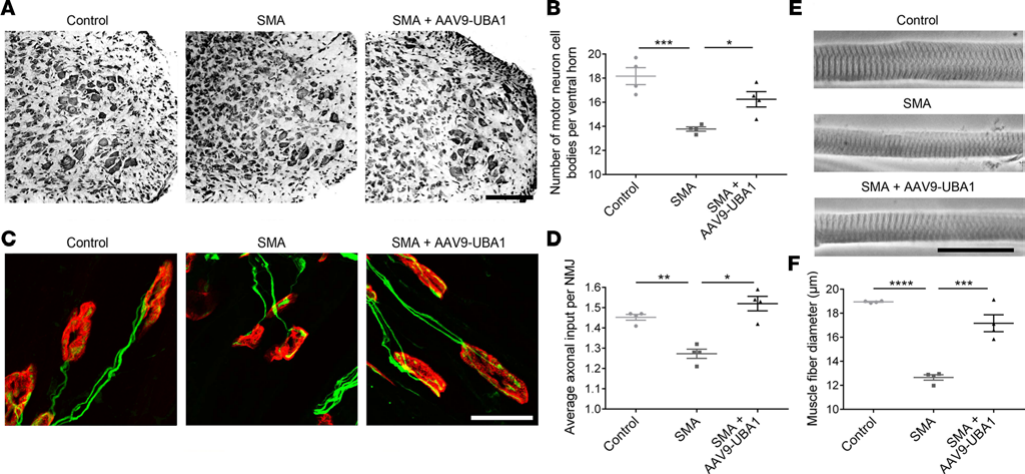
Rescue of neuromuscular and systemic pathology in SMA mice treated with AAV9-UBA1. (**A**) Nissl-stained spinal cords (ventral horn) from uninjected control, uninjected spinal muscular atrophy (SMA), and adeno-associated virus serotype 9–ubiquitin-like modifier activating enzyme 1–treated (AAV9-UBA1–treated) SMA mice at P9 (scale bar: 250 μm). (**B**) Significant rescue of motor neurons in AAV9-UBA1–treated SMA mice at P9 (*n* = 4 mice per group). (**C**) Confocal micrographs of neuromuscular junctions (NMJs) in the external oblique from uninjected control, uninjected SMA, and AAV9-UBA1–treated SMA mice at P9 (axonal inputs in green; postsynaptic endplates in red; scale bar: 25 μm). (**D**) Significant improvement in NMJ pathology in the external oblique muscle of AAV9-UBA1–treated SMA mice at P9 (*n =* 4 mice per group). (**E**) Bright-field micrographs of individual teased muscle fibers from the external oblique of uninjected control, uninjected SMA, and AAV9-UBA1–treated SMA mice at P9 (scale bar: 100 μm). (**F**) Significant rescue of muscle fiber diameters in AAV9-UBA1–treated SMA mice at P9 (*n =* 4 mice per group). One-way ANOVA with Tukey’s post-hoc test for all analyses. *P ≤ 0.05, **P ≤ 0.01, ***P ≤ 0.005, ****P ≤ 0.001. As part of a different comparative analysis, uninjected littermate control data motor neuron cell body counts (**B**) and NMJ axonal input (**D**) is also shown in Figure 2, F and H, respectively.

**Figure 6:**
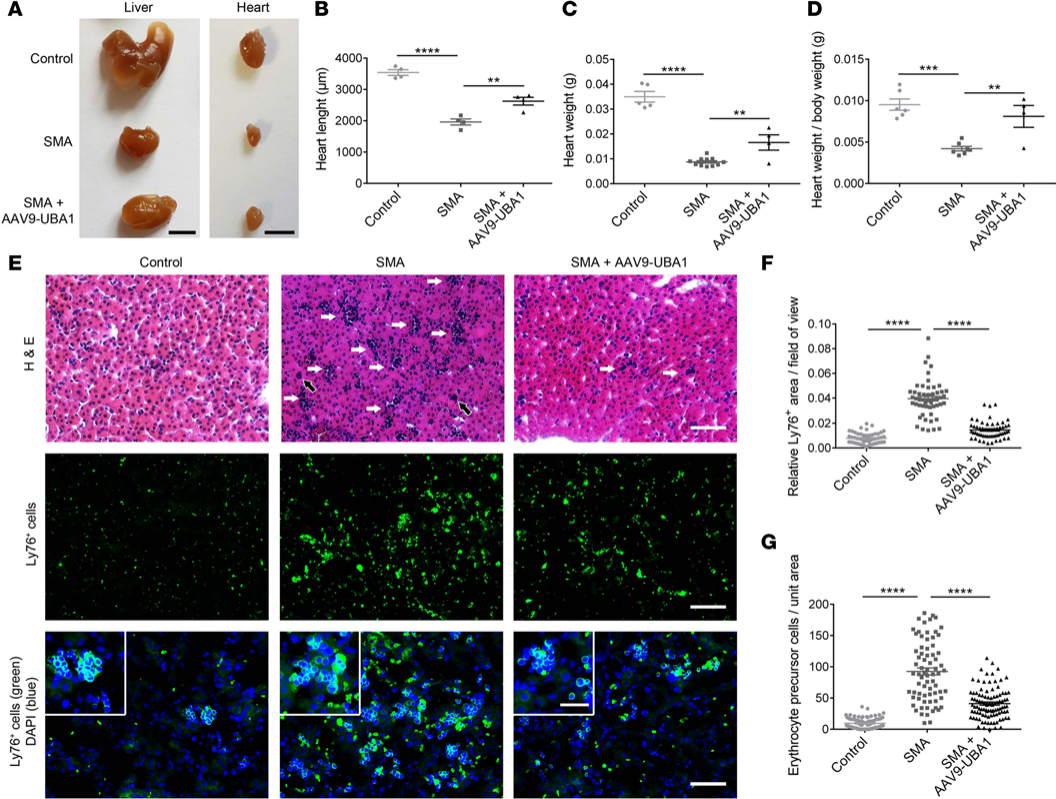
Rescue of heart and liver pathology in SMA mice treated with AAV9-UBA1. (**A**) Uninjected control, uninjected spinal muscular atrophy (SMA), and adeno-associated virus serotype 9–ubiquitin-like modifier activating enzyme 1–treated (AAV9-UBA1–treated) SMA mouse hearts and livers at P9 (scale bar: 0.5 cm). (**B**–**D**) Significant improvement in heart pathology in P9 AAV9-UBA1–treated SMA mice for the parameters of (**B**) heart length (*n =* 4 mice per group), (**C**) heart weight (control *n* = 5, SMA *n* = 11, SMA + AAV9-UBA1 *n* = 4), and (**D**) heart-weight-to-body-weight ratio (control *n* = 5, SMA *n* = 7, SMA + AAV9-UBA1 *n* = 4). (**E**) Histological analysis of uninjected control, uninjected SMA, and AAV9-UBA1 SMA P9 livers. Top row shows H&E-stained micrographs (white arrows indicate hematopoietic islands; black arrows indicate megakaryocytes; scale bar: 25 μm). Middle row shows micrographs of Ly76 immunohistochemistry (scale bar: 25 μm). Bottom row shows micrographs (scale bar: 25 μm) along their magnified insets (scale bar: 12.5 μm) costained with the Ly76 marker (green) and DAPI (blue). (**F** and **G**) Significant improvements in (**F**) liver Ly76^+^ erythrocyte area and (**G**) liver erythrocyte precursor cell area of AAV9-UBA1–treated SMA mice at P9 (*n =* 4 mice per group). One-way ANOVA with Tukey’s post-hoc test for all analyses. **P ≤ 0.01, ***P ≤ 0.005, ****P ≤ 0.001.

Given that AAV9-UBA1 treatment had a beneficial effect on the gross phenotype of SMA mice, we next analyzed the effect of increasing UBA1 levels on late-stage (P9) neuromuscular and organ pathology. In the neuromuscular system, AAV9-UBA1–treated SMA mice displayed a significant improvement in spinal motor neuron survival (Figure 5, A and B), a reversal of NMJ pathology (Figure 5, C and D, and Supplemental Figure 4, A and C), and restored muscle fiber diameters (Figure 5, E and F, and Supplemental Figure 4, B and D). The gross appearance of the heart was noticeably improved in AAV9-UBA1–treated SMA mice (Figure 6A), as reflected in an increased heart length (Figure 6B), heart weight (Figure 6C), and heart-weight-to-body-weight ratio (Figure 6D). H&E analysis of liver pathology in SMA mice revealed an increased number of nucleated cells and megakaryocytes (Figure 6E). Ly76/DAPI immunohistochemistry showed that SMA mouse livers have an accumulation of erythrocytes and nucleated erythrocyte precursors (Figure 6, E–G). AAV9-UBA1 treatment ameliorated megakaryocyte numbers (Figure 6E), Ly76^+^ erythrocyte accumulation (Figure 6, E and F), and nucleated Ly76^+^ erythrocyte precursor cell numbers (Figure 6, E and G), indicating a rescue of liver cellular pathology.

### AAV9-UBA1 treatment corrects expression of downstream UPS/UBA1 targets and increases SMN levels in SMA mice.

**Figure 7:**
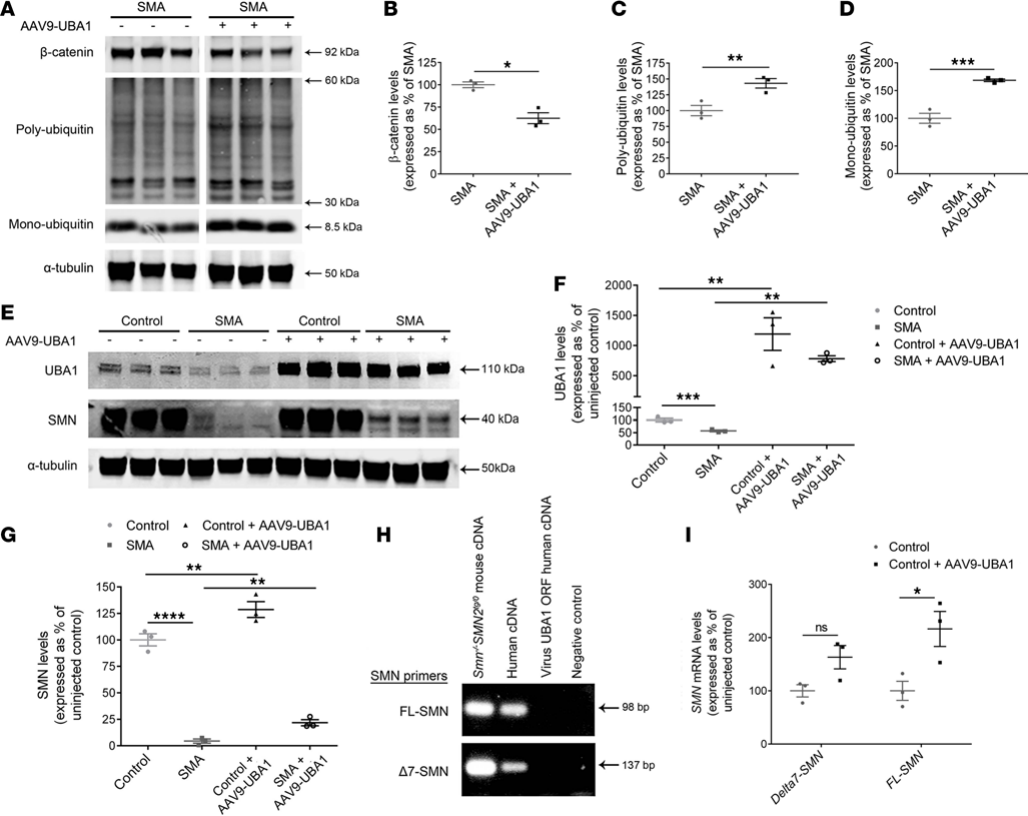
AAV9-UBA1 gene therapy corrects UPS perturbations and increases *FL-SMN* mRNA and protein levels. (**A**–**D**) Western blot analysis of β-catenin, polyubiquitin, and monoubiquitin protein levels in uninjected spinal muscular atrophy (SMA) and adeno-associated virus serotype 9–ubiquitin-like modifier activating enzyme 1 (AAV9-UBA1) SMA mouse hearts at P7 (*n =* 3 mice per treatment group; unpaired 2-tailed Student’s *t* test). Lanes were run on the same gel but were noncontiguous. (**E**–**G**) Western blot analysis of UBA1 and survival motor neuron (SMN) protein levels in uninjected control, uninjected SMA, AAV9-UBA1–treated control, and AAV9-UBA1–treated SMA P7 hearts (*n =* 3 mice per group; 1-way ANOVA with Tukey’s post-hoc test). (**H**) PCR products following mouse, human, and UBA1 viral cDNA amplification using primers that detect full-length *SMN* (*FL-SMN*) (top row) or *Δ7-SMN* (bottom row). (**I**) Significant increase in *FL-SMN*, but not *Δ7-SMN*, mRNA expression in the hearts of AAV9-UBA1–treated mice at P7, as detected by qRT-PCR quantification using the primers shown in **H** (*n =* 3 mice per treatment group; unpaired 2-tailed Student’s *t* test). ns (not significant) *P >* 0.05*,* *P ≤ 0.05, **P ≤ 0.01, ***P ≤ 0.005, ****P ≤ 0.001.

Next, we wanted to establish whether AAV9-UBA1 therapy was generating phenotypic improvements in SMA by correcting molecular perturbations known to occur downstream of UBA1 disruption in SMA. As the heart had the highest fold changes in UBA1 protein levels following gene therapy delivery (Figure 2, A and B, and Figure 3, B and C), we chose to conduct these investigations in heart tissue from control and SMA mice. Western blot analyses of a panel of proteins known to be perturbed in SMA mice as a consequence of reduced Uba1 levels (14) revealed a correction in levels of β-catenin as well as restoration of monoubiquitin and polyubiquitin in SMA mice treated with AAV9-UBA1 (Figure 7, A–D).

Given that SMN degradation is predominately regulated by the UPS (9), we next wanted to establish whether AAV9-UBA1 treatment resulted in altered levels of SMN. Western blot analysis revealed that higher levels of UBA1 protein in both control and SMA mice (Figure 7F) resulted in a significant increase in levels of SMN protein (Figure 7, E and G). No change in the levels of either UBA1 or SMN protein was detected in control or SMA mice that received an intravenous injection of AAV9-GFP (Supplemental Figure 5, A–D), indicating that the SMN increase in AAV9-UBA1–treated tissue was occurring as a direct result of UBA1 overexpression, rather than as a secondary consequence of AAV injection. qRT-PCR quantification of *Δ7-SMN* and full-length *SMN* (*FL-SMN*) mRNA expression in AAV9-UBA1–treated SMA mice revealed that, while *Δ7-SMN* expression was not significantly changed, *FL-SMN* mRNA levels were increased compared with uninjected controls (Figure 7, H and I). Thus, increased levels of UBA1 resulting from AAV9-UBA1 delivery were likely influencing SMA pathology via correcting defects in known UPS/UBA1 targets (e.g., β-catenin and ubiquitin homeostasis) as well as through modestly increasing levels of full-length SMN protein.

## Discussion

Recent studies have identified widespread dysregulation of ubiquitin homeostasis as a conserved molecular hallmark of SMA. Here, we demonstrate that ubiquitin pathway defects represent a key driver of both neuromuscular and systemic pathology in SMA, with targeting of UBA1 representing a safe and effective approach for developing novel and effective SMN-independent therapies. Our finding that UBA1 suppression occurred in iPSC-derived human SMA motor neurons, with a similar magnitude of response to that previously reported in animal models (14), confirms that UBA1 represents a valid and attractive therapeutic target for clinical translation. The subsequent demonstration that restoration of Uba1 in zebrafish generated a robust rescue of functional and morphological neuromuscular defects therefore represents an important proof-of-concept demonstration that targeting UBA1 can be sufficient to ameliorate disease pathogenesis in SMA. Taken together with our detailed AAV9-UBA1 safety and efficacy studies in mice, this provides support for the notion that UPS perturbations in SMA are a clinically relevant molecular defect representing a viable therapeutic target in human patients.

The demonstration that elevated levels of UBA1 resulting from treatment with AAV9-UBA1 were safe and well tolerated in vivo (both in healthy and SMA mice) is particularly encouraging. This may be particularly pertinent as gene mutations or alterations in UBA1 expression have been associated with cell proliferation (24) and cancer (25, 26). Reassuringly, no obvious signs of gross tumor formation were noted over a 6-month period in control mice treated with AAV9-UBA1, consistent with previous work showing that syngeneic transplantation of UBA1-overexpressing melanoma cells did not significantly affect tumor formation in mice (25). This increased our confidence in our ability to selectively target and correct defective aspects of the UPS in SMA (in this case, not only rectifying UBA1 suppression, but also consequently correcting monoubiquitin and polyubiquitin levels as well as downstream substrate targets such as β-catenin) without destabilizing the entire system or generating unintended phenotypes. The fact that high levels of UBA1 are well tolerated is perhaps not too surprising, considering that UBA1 is in the top 2% of the most abundant cellular proteins (27), suggesting that cells already have the capacity to handle high levels of this important protein.

Our finding that AAV9-UBA1 gene therapy was able to rescue neuromuscular pathology in SMA mice, with associated improvements in body weight, survival, and motor performance, confirms that targeting of UBA1 represents a valid approach to therapy development for SMA (alone or in combination with other SMN-targeted therapies). Systemic pathology in the heart and liver were also ameliorated following AAV9-UBA1 delivery. The liver phenotype described here, whereby greater numbers of hematopoietic elements such as erythrocyte precursor cells and megakaryocytes are seen due to SMN depletion, resembles that of liver conditional SMN knockout mice (28). The liver is the main hematopoietic organ during embryonic development, but this function is rapidly lost in the early postnatal period as bone marrow takes over (29). As bone development in SMA is impaired (30), it may therefore be unable to support hematopoiesis, leading to persistent erythropoiesis in the liver. As this pathology is ameliorated by UBA1 overexpression, it seems likely that such changes in hematopoiesis are due to aberrant rather than delayed liver development.

The robust neuromuscular and systemic phenotypic improvements we report are perhaps even more striking when viewed in light of the fact that we were restricted to the use of “standard” (i.e., not self-complimentary) AAV9 vectors, resulting in a slower onset of UBA1 expression than we might have otherwise desired. This point is particularly important to note in light of several recent studies demonstrating that SMN-dependent therapies have a very narrow time window of effectiveness (essentially the first few days of life) in SMA mice (31–33). Moreover, the increased survival we obtained using AAV9-UBA1 in the severe “Taiwanese” SMA mouse model (with a life expectancy of ~9 days in our hands at present) is very similar in magnitude to the increase in survival previously reported in studies restoring SMN with AAV9 in comparable severe SMA mouse models (34) (in contrast to studies using the milder Δ7 SMA mouse model). Thus, next-generation therapeutic strategies capable of delivering a more rapid targeting of UBA1 levels and/or activity may have an even better potential to modify core disease parameters in SMA. In addition, it is tempting to speculate that combined delivery of UBA1-targeted therapies alongside SMN-targeted therapies may offer the potential to generate a more robust, systemic improvement in disease symptoms than either UBA1- or SMN-directed treatments alone. Furthermore, the UBA1-targeted therapies we report here may represent novel treatment options for patients with X-linked SMA due to mutations in the *UBA1* gene (17–19).

Molecular analysis of AAV9-UBA1–treated SMA mouse hearts showed that the virally delivered human UBA1 gene was functional in mice in vivo, with increased UBA1 levels sufficient to correct downstream UPS perturbations, reducing β-catenin and increasing monoubiquitin and polyubiquitin levels previously identified in SMA mice (14). This correction of downstream UPS perturbations is likely to contribute directly to the improved motor performance of SMA mice following UBA1 gene therapy, as inhibition of the UBA1 downstream target β-catenin has previously been shown to improve neuromuscular phenotypes in the same SMA mouse model (14). In addition, examination of SMN levels in the treated mice revealed that increasing UBA1 levels resulted in modestly increased expression of *FL-SMN* mRNA and SMN protein in vivo. Although the magnitude of increase in SMN expression is unlikely to explain all the phenotypic improvements observed in SMA mice following UBA1 gene therapy, it is likely to be contributing, at least in part, to the amelioration of disease symptoms. While it is known that the UPS regulates SMN degradation (8, 9), our findings suggest that further work is now required to fully determine the specific role of UBA1 in this process.

Our current findings, taken together with previous studies showing that loss-of-function mutations in *UBA1* cause X-linked SMA (17–19) and that *Uba1* loss-of-function *Drosophila* (35–37) and *C*. *elegans* (38) mutants display motor impairments, provide a direct demonstration that UBA1 is required for maintenance of a healthy neuromuscular system and for normal neuronal function. Similarly, emerging evidence suggests that UBA1 dysregulation may be implicated in other neurodegenerative diseases (39) and may also play a role in neuroprotection (for example, UBA1 levels are increased in neurons from *Wld^s^* mice protected from Wallerian degeneration; refs. 40, 41). Thus, the development of UBA1-targeted therapies for SMA reported here may have potential relevance to a broad range of other neurodegenerative conditions.

## Methods

### Zebrafish Smn knockdown and UBA1 restoration.

*Smn* morpholino oligonucleotides (MO) were designed against the 5′ start sequence of the *Smn* gene (Gene Tools LLC), 5′-CGACATCTTCTGCACCATTGGC-3′ (42). Full-length human *UBA1* (NM_003334.3) was ligated into a PCS2+MT vector using BamH1 restriction sites. The *UBA1* construct was used to generate *UBA1* mRNA for overexpression experiments. Single-cell stage *Tg(mnx1:GFP ^ml2^)* embryos (43) were injected with 4 ng *Smn* MO in aqueous solution containing 0.05% phenol red or 4 ng *Smn* MO coinjected 200 ng/μl human *UBA1* mRNA in aqueous solution. Uninjected zebrafish were used as controls. Zebrafish embryos and litters of mice were randomly assigned to treatment groups. Unfertilized or severely deformed embryos were excluded from analysis. Sample size estimates were based on previous analyses using these models (14).

### Zebrafish motor axon phenotype analysis.

For immunostaining, uninjected *Smn* MO and *Smn* MO + UBA1 *Tg(mnx1:GFP ^ml2^)* embryos were dechorinated at 30 hours after fertilization and fixed in 4% PFA overnight before being dehydrated in 100% methanol. Embryos were rehydrated and washed in PBS before being transferred into 100% acetone for 10 minutes at –20°C for permeabilization. Embryos were washed in PBST (PBS, 1% DMSO, 1% BSA and 0.5% Triton-X 100) before being blocked in PBST plus 2% sheep serum, followed by overnight incubation of chicken anti-GFP primary antibody (1:1,000; Abcam; ab13970). Embryos were washed in PBST and then incubated in Alexa Fluor 488 secondary antibody (1:500; Jackson ImmunoResearch; 703-545-155). Embryos were mounted in 80% glycerol and imaged at ×20 objective using a Zeiss LSMZ10 confocal microscope. The percentage of motor neurons with normal axons, branched axons, and severely abnormally truncated axons was then analyzed as previously described (14) (*n =* 20 per group, 12 axons per embryo [6 segments] analyzed behind the yolk). Motor axon analysis was performed by investigators blinded to the treatment group.

### Zebrafish behavioral testing.

Uninjected (*n =* 18), *Smn* MO (*n =* 33), and *Smn* MO + UBA1 (*n =* 32) *Tg(mnx1:GFP ^ml2^)* embryos were left to develop until 3 days after fertilization before behavioral analysis. Three days after fertilization, embryos were touched with a glass capillary on the tail end. Their swim path was recorded over 15 seconds and analyzed using a Noldus behavior analysis setup and EthoVision software (version 7). The swim arena was divided into quadrants, and the data were analyzed based on how many quadrants the embryos entered. Recordings of each embryo were performed 3 times, and the mean was taken.

### Preparation of zebrafish for Western blot analysis.

Wild-type AB strain 1-cell stage embryos were injected with 6 ng of *smn* MO and left to develop until 48 hours after fertilization. Embryos were dechorinated and deyolked in 1 ml of deyolking buffer (1/2 Ginzburg Fish Ringer without calcium: 55 mM NaCl, 1.8 mM KCl, 1.25 mM NaHCO_3_). Embryos were pooled into batches of 30 fish, with 3 replicate batches per experimental group. Zebrafish were pelleted at 300 *g* for 30 seconds, and the supernatant was discarded. Zebrafish were washed twice with wash buffer (110 mM NaCl, 3.5 mM KCl, 2.7 mM CaCl_2_, 10 mM Tris, pH 8.5) and cells were pelleted at 300 *g* for 30 seconds. The supernatant was removed, and cells were stored at –80°C.

### SMA patient and control iPSC-derived motor neurons.

iPSC-derived motor neurons were generated by reprogramming Coriell Biorepository patient fibroblasts as previously described (20) (*n =* 8 independent clones of control iPSC motor neurons were generated from fibroblast line GM03814 and *n =* 9 independent SMA clones generated from fibroblast lines GM03813 or GM00232). Western blot analysis was performed on cells that were harvested at 14 days after differentiation.

### AAV production.

AAV9-UBA1 and AAV-GFP were produced as previously described (44). Briefly, HEK293T cells cultured in 15-cm dishes were transfected with 26 μg Helper plasmid (pHelper) (Stratagene), 13 μg packaging plasmid (pAAV2/9) (provided by J. Wilson, University of Pennsylvania, Philadelphia, Pennsylvania, USA), and 13 μg pAAV-CMV-UBAI plasmid using polyethylenimine (PEI, linear, MW 25,000) (Polysciences Inc., 23966) transfection method. Five days after transfection, the supernatant was collected and treated with Benzonase nuclease (Sigma-Aldrich, E1014-25KU) for 2 hours at 37°C. The supernatant was then centrifuged (3,850 *g* for 5 minutes) in order to be clarified from cell debris before being filtered using a 0.22-μM vacuum filter. It was then concentrated by approximately 75 times using Amicon Ultra-15 Centrifugal 100K filter units. AAV purification by iodixanol density gradient and collection of the 40% layer containing the virus was performed following concentration. Purified, concentrated, and desalted AAV vector was then titered using qPCR. The vector map for AAV-UBA1 containing the full-length human UBA1 open reading frame (NM_003334.3) is shown in Supplemental Figure 1.

### Mouse model of SMA.

The “Taiwanese” mouse model of severe SMA (*Smn^-/-^; SMN2^tg/0^*) on a congenic FVB background was used throughout (45), utilizing a previously described breeding strategy (46). Age-matched, phenotypically normal littermate mice (*Smn^+/-^; SMN2^tg/0^*) were used as controls, and litters were retroactively genotyped using standard PCR protocols (46). Mice were originally obtained from Jackson Laboratories and were maintained in animal care facilities at the University of Edinburgh under standard specific pathogen–free conditions. CD1 mice were used as wild-type animals for AAV9-GFP biodistribution analyses.

### AAV9 gene therapy delivery.

For all gene therapy analysis, data from uninjected littermate controls, AAV9-UBA1 littermate controls, uninjected SMA mice, and AAV9-UBA1 injected mice was obtained from the same set of experiments. For subsequent analysis, we present different comparisons between these 4 experimental groups, hence the repeated presentation of data for the uninjected control experimental group in some figures.

An initial biodistribution study assessed the spread of Evans blue dye (1% w/v; Sigma-Aldrich) following delivery of a 10-μl injection volume to the intraperitoneal cavity or intravenously via the facial vein. As intravenous injections were found to be superior at achieving systemic distribution, all subsequent delivery of gene therapy was given via injection into the facial vein in TTG or CD1 wild-type mice, conducted under chilled anesthesia using a 33-gauge needle and Hamilton syringe (Supplemental Figure 2). AAV9-UBA1 was administered at the day of birth (P1) at a concentration of 2.4 × 10^11^ vg/mouse and AAV9-GFP was administered at a concentration of 1.4 × 10^11^ vg/mouse at a 10-μl injection volume. Visualization of the facial vein for intravenous injection was aided by using a Wee Sight Transilluminator LED light (Philips) (Supplemental Figure 2). Successful intravenous injection was noted by blanching of the vein during injection. Bleeding at the injection site was minimized by applying pressure with a tissue. Pups were held in the hand until their temperature and movement returned to normal; then they were placed into a cage with a CD1 foster mother. Uninjected mice were used as controls. Whole litters of mixed male/female pups were used. Litters of mice were randomly assigned to treatment groups. Sample size estimates were based on previous analyses using these models (14). Whole litters of mice were excluded from the study if there was evidence of clear maternal neglect (e.g., cannibalization or lack of feeding).

### Weights, survival, and righting test.

Mice assigned to each of the treatment groups were weighed on a daily basis. For long-term analysis of uninjected and AAV9-UBA1–treated controls, mice were weighed on a monthly basis. Kaplan-Meier survival analyses were performed on uninjected or AAV9-UBA1–treated mice as previously described (47). For SMA mice, a predetermined endpoint of survival was based on an evaluation of their established and robust clinical phenotype (14) and/or weight loss of 20% from the peak body weight of that animal, persisting for 72 hours. To assess motor performance of uninjected or AAV9-UBA1–treated mice, a righting reflex was performed daily as previously described (48). For each daily session, the righting reflex test was performed 3 times and the mean was taken.

### Neuromuscular analysis.

Analysis of neuromuscular pathology was performed on uninjected control, uninjected SMA, control + AAV9-UBA1, and SMA + AAV9-UBA1 P9 mice (*n =* 4 for each group). Spinal motor neuron cell body counts were performed as previously described (15). NMJ pathology was performed on whole-mount external oblique and transversus abdominis muscles as previously described (49). Example images were taken using a Nikon A1R confocal system combined with a Ti:E inverted microscope (×60 objective). Muscle fiber diameter measurements were taken from phase-contrast micrographs of external oblique and transversus abdominis muscle preparations using ImageJ software (NIH) as previously described (50). Bright-field images of spinal motor neurons and muscle fiber diameters were taken using a Leica DMIRB microscope equipped with a Retiga 2000R camera and QCapturePro-6 software (×20 objective). Neuromuscular analysis was performed by investigators blinded to the treatment group.

### Heart and liver analysis.

Freshly dissected hearts from P9 uninjected control (*n =* 5), uninjected SMA (*n =* 11) and SMA + AAV9-UBA1 (*n =* 4) mice were immediately weighed using a microbalance. The heart-weight-to-body-weight ratio was calculated by dividing the weight of the freshly dissected hearts by the whole-body weight for each animal. Heart length was determined by cyrosectioning the whole length of heart from apex to base. Each 12-μm heart section was collected on to slides and processed by H&E staining, and the sum of total section number per heart was multiplied by 12 to give the length in μm. Analysis of liver pathology in P9 uninjected control, uninjected SMA, and SMA + AAV9-UBA1 mice (*n =* 4 mice for each group) was performed using H&E staining, Ly76, and DAPI immunohistochemistry (details below). For microstructural analysis, livers fixed in 4% paraformaldehyde were transferred to 70% ethanol and wax embedded before being cut at 3 μm on a microtome and stained with H&E using a standard protocol. Sections were visualized using a Nikon eclipse e400 microscope (×10 objective), and images were captured using a QICAM Fast 1394 camera and Improvision Velocity 4 image capture software. Ly76 density quantification was performed using ImageJ (NIH). Cells that were positive for Ly76 and DAPI were classified as erythrocyte precursor cells.

### Hematology and serum biochemistry analysis.

Under terminal anesthesia, blood was collected via cardiac puncture from uninjected P30 control littermate mice or P30 control mice that had received an AAV9-UBA1 injection at P1 (*n =* 3 per group). Blood was collected into heparin- and EDTA-coated tubes, kept on ice, and immediately taken for further processing. Hematology analysis was performed using a ABX Pentra 60 hematology analyser (Horiba Ltd.), with the differential white blood cell counts and differential done manually (100-cell count) on blood smears stained with modified Wright’s stain. Analysis of serum biochemistry was performed using an IL650 biochemistry analyser (Diamond Diagnostics) with assay kits from Randox Ltd. (Crumlin).

### Quantitative fluorescent Western blot analysis.

Tissue for Western blot analysis was quickly dissected, placed on dry ice, and stored at –80°C. Protein was extracted in RIPA buffer (ThermoScientific) with 1% protease inhibitor cocktail (Sigma-Aldrich), and the protein concentration was then determined by BCA assay (ThermoScientific). SDS-PAGE was performed using precast NuPage 4%–12% BisTris gradient gels (Life Technologies) and transferred to PVDF membranes by the iBlot 7 minute semi-dry blotting system (Life Technologies). Following Ponceau S stain and blocking, membranes were incubated overnight at 4°C using the following primary antibodies: mouse anti-ubiquitin (clone Uba-1) (1:1,000, Merck Millipore, MAB1510), mouse anti-SMN (1:1,000, BD Biosciences, 610646), mouse anti–β catenin (1:1,000, BD Biosciences, 610153), rabbit-anti UBE1a,b (1:1,000, ThermoFischer, PA5-17274), rabbit anti-E1 ubiquitin activating enzyme (1:1,000, Abcam, ab34711), rabbit anti-GFP (1:2,000, Abcam, ab290), mouse anti–α tubulin (DMA1) (1:10,000, Abcam, ab7291), rabbit anti–β-III tubulin (Abcam, 1:10,000, 18207), and anti-COX IV (20E8C12) (1:1,000, Abcam, ab14744). Following PBS washes, the following secondary antibodies were used: IR dye 800CW goat anti-mouse IgG (926-32210), IR dye 800CW goat anti-rabbit IgG (926-3211), IR dye 680RD goat anti-mouse IgG (926-68070), and IR dye 680RD goat anti-rabbit IgG (926-68071), all at 1:5,000 and from LI-COR Biosciences. Membranes were imaged using an Odyssey Infrared Imaging System (Li-COR, Biosciences) and quantified using Image Studio software (Li-COR). Band intensities were then normalized to a loading to control to determine final relative protein expression.

### Immunohistochemistry.

Tissue from uninjected and gene therapy–treated mice was dissected and fixed in 4% paraformaldehyde in PBS for 4 hours at 4°C before being transferred into 30% sucrose solution overnight at 4°C for cryoprotection. Tissue was embedded in OCT and using a cryostat, where heart (20 μm), liver (20 μm for GFP and UBA1 and 7 μm for Ly-76), and spinal cord (25 μm) sections were collected on polysine-coated slides (Thermo Scientific). Sections were permeabilized in 0.3% Triton X-100 in PBS and then blocking solution (4% BSA, 0.3% Triton X-100 in PBS) for 30 minutes at room temperature before overnight incubation with primary antibody solution at 4°C: rabbit anti-GFP (1:500, Abcam, ab290), mouse anti-UBA1 (1:200, Sigma-Aldrich, E3125), and rat anti-Ly76 (1:100, Abcam, ab91113). After PBS washes, sections were incubated with secondary antibody solution for 2 hours at room temperature: Alexa Fluor 488 donkey anti-rabbit IgG (1:500, Life Technologies, A-21206), Alexa Fluor 488 donkey anti-mouse IgG (1:500, Life Technologies, A-21202), and Alexa Fluor 488 goat anti-Rat IgG (1:200, Life Technologies A-11006). Sections were counterstained with Neurotrace 435/455 blue fluorescent Nissl stain (1:100, Life Technologies, N21479) or DAPI nuclei stain (1:1,000, Life Technologies, D1306) for 10 minutes. For GFP and UBA1 immunohistochemistry, images were taken using a Nikon A1R confocal system combined with a Ti:E inverted microscope (×40 objective). For Ly76 liver analysis, images were taken using a Nikon eclipse e400 microscope (×10 objective) with a QICAM Fast 1394 camera and Improvision Velocity 4 image capture software.

### qRT-PCR.

Extraction of total RNA from hearts of P7 uninjected and AAV9-UBA1–treated control mice (*n =* 3 mice for each treatment group) was achieved by using a RNAeasy Mini kit (Qiagen). Human blood cDNA was a gift from Kathy Evans, University of Edinburgh. Samples were checked for DNA contamination and RNA concentration was determined by a Nanodrop 2000 spectrophotometer (Thermo Scientific). RNA integrity was checked by resolution of two strong 28S and 18S rRNA bands in a ratio of 2:1 by agarose gel electrophoresis. cDNA was made from 1.5 μg RNA using a High Capacity cDNA Reverse Transcription kit (Life Technologies). Primers were designed that amplified transcripts unique to mouse *Uba1* (forward CCTACATGACCAAGGAACTA, reverse AGCTGTTGAGTTCAGCAAGT), human *UBA1* (forward TCTGTGCTCAGCATGGCCGG, reverse ATGAGGTCCTCGTCCAGGTTA), or a conserved sequence in both mouse *Uba1* and human *UBA1* (forward TGTCCAAGAAACGTCGCG, reverse CTCGTCTATGTCTGCTTCACTG). *FL-SMN* and Δ7 *SMN* primer sequences were taken from the published literature (51). qPCR reactions were performed with a Bio-Rad CFX Connect Optis module real-time system thermocycler (Bio-Rad). For all primer pairs, cDNA was amplified in triplicate using KAPA SYBR FAST qPCR Master Mix (KAPA Biosystems) using a standard PCR program. Samples with Cq values >35 cycles were considered as having no detectable expression. A gene study using qPCR analysis was performed using Bio-Rad CFX manager 3.1 software where relative expression levels was normalized using the geometric mean of 3 housekeeping genes: *PPIA* (forward GCGTCTCCTTCGAGCTGTTTG, reverse TGAAAGTCACCACCCTGGCACAT), *GAPDH*, and *OAZ1* (sequences previously described; see ref. 14).

### Statistics.

Data were collected and analyzed using Microsoft Excel and GraphPad Prism 6 software. Individual statistical tests are described in the main text and figure legends. For Student’s *t* tests, an unpaired, 2-tailed analysis was performed. For all analysis, *P* ≤ 0.05 was considered statistically significant. All data are expressed as mean ± SEM.

### Study approval.

All experimental procedures involving animals were conducted in accordance with United Kingdom Home Office regulations and were approved by a University of Edinburgh internal ethics committee and veterinary staff.

## Author contributions

THG, MA, and TMW conceived the study; RAP, EK, PB, JC, CM, RAJ, YZ, ES, EJNG, GH, DT, CGB and THG designed and carried out the experimental work; and RAP, PB, RAJ, YZ, ES, SHP, and THG analyzed data. All authors contributed to the writing of the manuscript.

## Supplementary Material

Supplemental data
