# Supplementary material for: Systemic restoration of UBA1 ameliorates disease in spinal muscular atrophy
Source: JCI Insight. 2016 Jul 21;1(11):e87908. doi: 10.1172/jci.insight.87908 (PMC5033939; doi:10.1172/jci.insight.87908)
Supplement: Supplemental data [file jciinsight-1-87908-s001.pdf]

**Supplemental data for:**

# Systemic restoration of UBA1 ameliorates disease in spinal muscular atrophy

Rachael A. Powis, Evangelia Karyka, Penelope Boyd, Julien Côme, Ross A. Jones, Yinan Zheng, Eva Szunyogova, Ewout J.N. Groen, Gillian Hunter, Derek Thomson, Thomas M. Wishart, Catherina G. Becker, Simon H. Parson, Cécile Martinat, Mimoun Azzouz, Thomas H. Gillingwater*

*To whom correspondence should be addressed: T.Gillingwater@ed.ac.uk

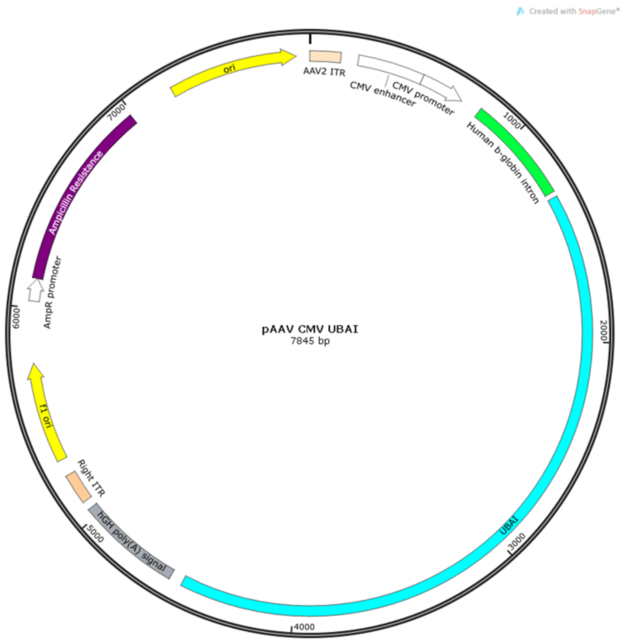


**Supplemental Figure 1. Vector map of the AAV9-CMV-UBA1 construct.**

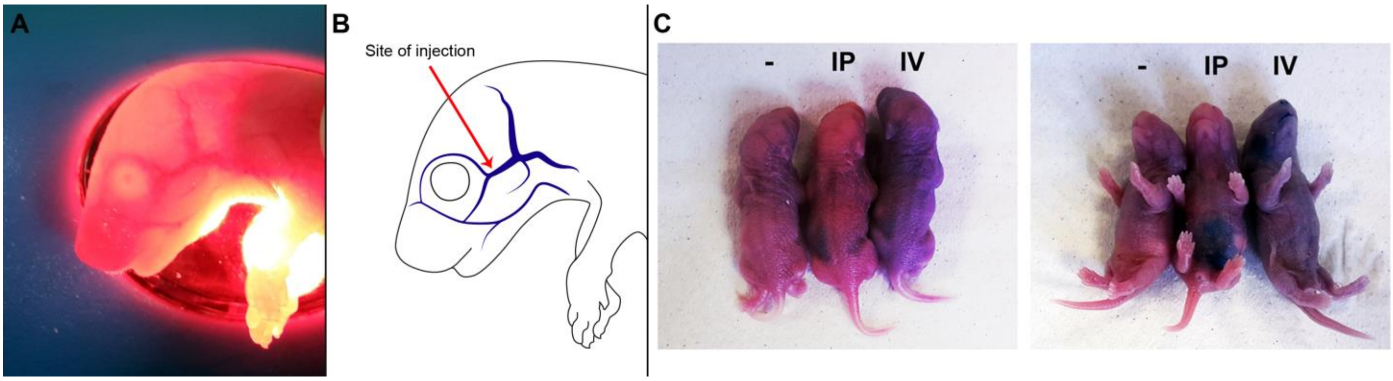


**Supplemental Figure 2. Facial vein injection of P1 mice leads to rapid and widespread systemic delivery.** (**A-B**) Photograph and diagram illustrating the site of adeno-associated virus serotype 9 (AAV9)-ubiquitin-like modifier activating enzyme 1 (UBA1) intravenous injection in P1 mice via the facial vein. (**C**) Photograph of P1 wild-type mice either uninjected (-) or following an injection of Evans Blue dye in the intraperitoneal cavity (IP) or intravenously via the facial vein (IV). Note the restricted localisation in the intraperitoneal cavity with IP injection compared to the widespread distribution with IV injection.

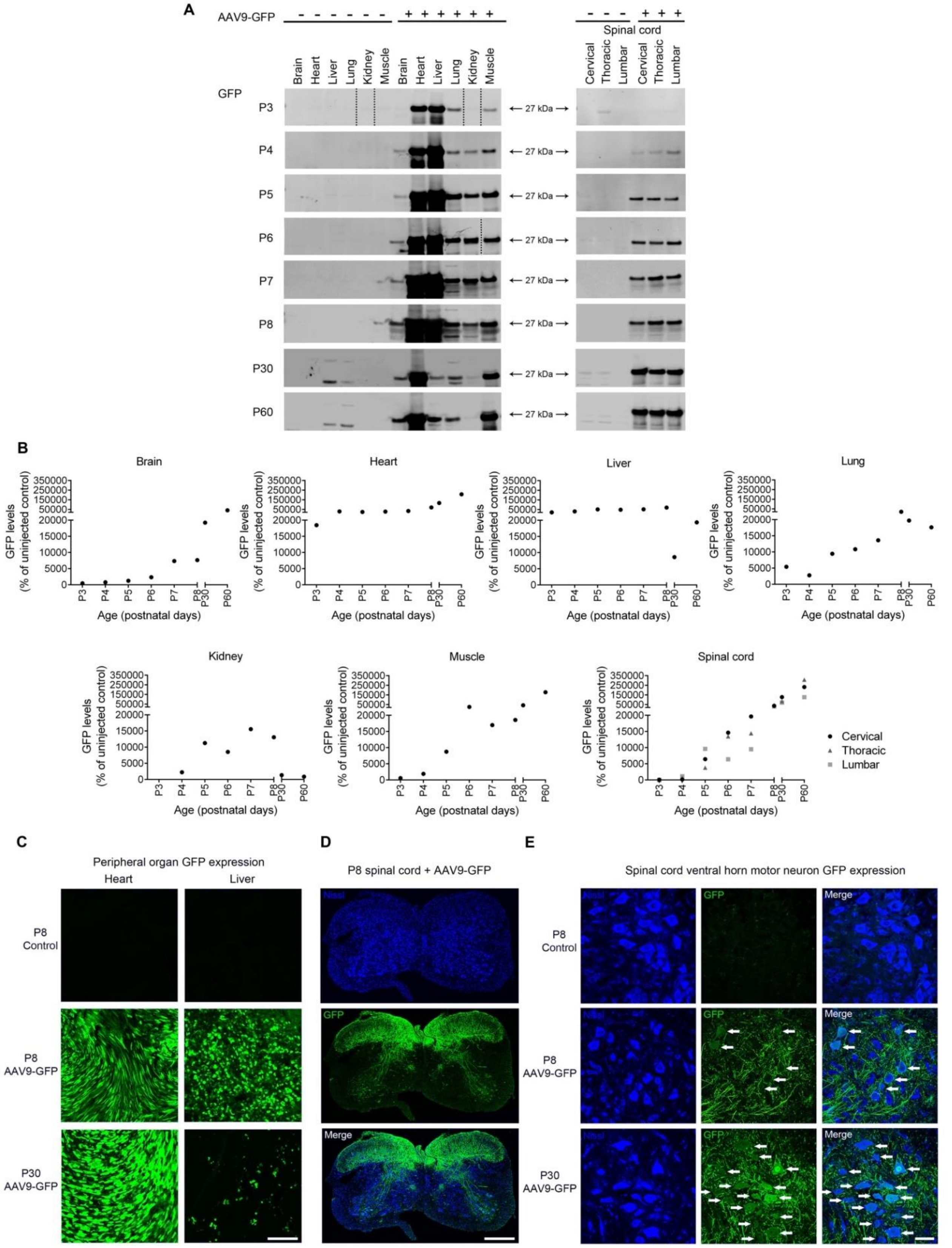


**Supplemental Figure 3. Bio-distribution of GFP expression in CD1 wildtype mice following an intravenous injection of AAV9-GFP at P1**. (**A-B**) Western blot analysis showing the presence and quantification of GFP protein levels in brain, heart, liver, lung, kidney, muscle and spinal cord (cervical, thoracic and lumbar levels) of wildtype CD1 mice culled at P3, P4, P5, P6, P7, P8, P30 and P6 following injection of adeno-associated virus serotype 9 (AAV9)-GFP via the facial vein at P1 (+) and uninjected controls (-), *n*=1 for each time point. Dotted lines represent image processing to align lane well order. (**C**) Representative confocal micrographs of GFP expression in the heart and liver of P8 uninjected CD1 mice and P8 and P30 mice which received an injection of AAV9-GFP via the facial vein at P1 as visualised using immunohistochemistry. Scale bar = 250 µm. (**D**). Representative confocal micrographs of GFP expression (green) in the L4-L5 lumbar spinal cord of a P8 wildtype mouse which had received an injection of AAV-GFP at P1. Spinal cords were stained with fluorescent Nissl (blue) (scale bar = 250 µm). (**E**). High magnification micrographs of the spinal cord ventral horn showing GFP expression (green) from P8 uninjected CD1 mice and P8 and P30 mice which received an injection of AAV9-GFP via the facial vein at P1. Spinal cords were co-stained with fluorescent Nissl (blue); white arrows indicate the presence of GFP in spinal motor neurons (scale bar = 50 µm).

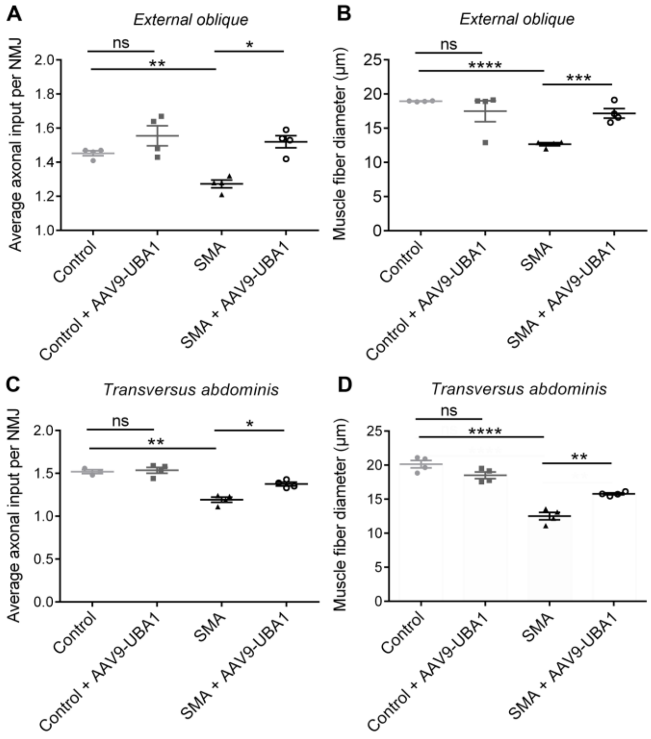


**Supplemental Figure 4. Rescue of muscle fiber diameter and NMJ pathology in the *External oblique* and *Transversus abdominis* muscles of SMA mice treated with AAV9-UBA1.** (**A-B**) Graphs showing (**A**) the mean axonal input per neuromuscular junction (NMJ) and (**B**) mean muscle fiber diameter in the *External oblique* of P9 uninjected control, adeno-associated virus serotype 9 (AAV9)-ubiquitin-like modifier activating enzyme 1 (UBA1) treated control, uninjected spinal muscular atrophy (SMA) and AAV9-UBA1 treated SMA mice. (**C-D**) Graphs showing (**C**) the mean axonal input per NMJ and (**D**) mean muscle fiber diameter in the *Transversus abdominis* of P9 uninjected control, AAV9-UBA1 treated control, uninjected SMA and AAV9-UBA1 treated SMA mice. For both muscles $n$=3 mice per treatment group; one-way ANOVA with Tukey's *post hoc* test. ns = not significant, * $P \leq 0.05$, ** $P \leq 0.01$, *** $P \leq 0.005$, **** $P \leq 0.001$.

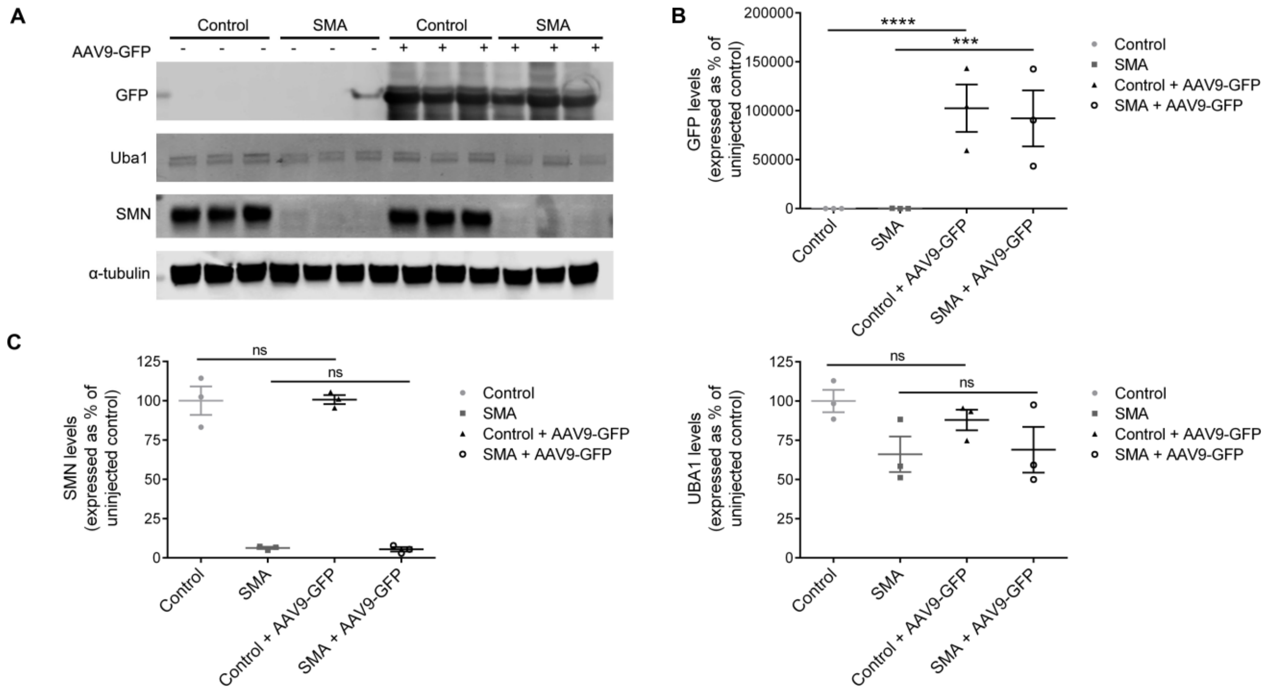


**Supplemental Figure 5. No change in UBA1 or SMN protein levels in control or SMA AAV9-GFP treated hearts.** (**A**) Western blot analysis showing GFP (top panel), ubiquitin-like modifier activating enzyme 1 (UBA1)(upper middle panel), and survival motor neuron (SMN) (lower middle panel) protein levels in the heart of P7 uninjected control, uninjected SMA, adeno-associated virus serotype 9 (AAV9)-GFP treated control and AAV9-GFP treated SMA mice. Bottom panel shows α-tubulin loading controls. (**B**-**D**) Quantification of GFP (**B**), UBA1 (**C**), and SMN (**D**) protein levels in the heart of P7 uninjected control, uninjected SMA, AAV9-GFP treated control and AAV9-GFP treated SMA mice (*n* = 3 for each treatment group; unpaired, two-tailed Student's *t*-test). ns = not significant, $P \leq 0.005$, **** $P \leq 0.001$.

**Supplemental Table 1.**

**Haematology analysis of blood from P30 uninjected control littermate mice and P30 control littermates which had received an intravenous injection of AAV9-UBA1 at P1.**

| Haematology | | | | |
|---|---|---|---|---|
| | **Units** | **Uninjected control** | **AAV9-UBA1** | ***P*-value** |
| White blood cells | x10^9/l | 1.9 ± 0.4 | 2.0 ± 0.2 | 0.876 |
| Neutrophils (segmented) | x10^9/l | 0.186 ± 0.049 | 0.145 ± 0.016 | 0.476 |
| Neutrophils (segmented) | % | 9.3 ± 0.9 | 7.3 ± 0.9 | 0.184 |
| Neutrophils (non-segmented) | x10^9/l | 0 | 0 | N/A |
| Neutrophils (non-segmented) | % | 0 | 0 | N/A |
| Lymphocytes | x10^9/l | 1.743 ± 0.328 | 1.85 ± 0.150 | 0.786 |
| Lymphocytes | % | 90.3 ± 0.6 | 92.3 ± 0.7 | 0.101 |
| Monocytes | x10^9/l | 0.004 ± 0.004 | 0.333 ± 0.333 | 0.379 |
| Monocytes | % | 0.3 ± 0.3 | 0 | 0.373 |
| Eosinophils | x10^9/l | 0 | 0 | N/A |
| Eosinophils | % | 0 | 0 | N/A |
| Basophils | x10^9/l | 0 | 0 | N/A |
| Basophils | % | 0 | 0 | N/A |
| Red blood cells | x10^12/l | 7.42 ± 0.42 | 6.98 ± 0.12 | 0.365 |
| Packed cell volume | l/l | 0.379 ± 0.022 | 0.361 ± 0.010 | 0.512 |
| Haemoglobin | g/dl | 12.4 ± 0.7 | 12.0 ± 0.3 | 0.625 |
| Mean corpuscular volume | fl | 51.0 ± 0.0 | 51.7 ± 0.9 | 0.492 |
| Mean corpuscular haemoglobin concentration | % | 32.8 ± 0.1 | 33.3 ± 0.4 | 0.277 |
| Red cell distribution width | % | 14.4 ± 0.4 | 13.8 ± 0.1 | 0.214 |

Data shown at mean ± SEM. *n* = 3 for each treatment group. Groups compared using an unpaired, two-tailed Student's *t*-test

**Supplemental Table 2.**

**Blood serum biochemistry analysis of blood from P30 uninjected control littermate mice and P30 control littermates which had received an intravenous injection of AAV9-UBA1 at P1.**

| Serum Biochemistry | | | | |
|---|---|---|---|---|
| | **Units** | **Uninjected control** | **AAV9-UBA1** | ***P*-value** |
| Albumin | g/l | 26.2 ± 1.5 | 25.4 ± 0.9 | 0.651 |
| Alanine aminotransferase | IU/L | 48 ± 3 | 52 ± 2 | 0.436 |
| Bile Acids | µmol/l | 1.1 ± 0.3 | 0.8 ± 0.4 | 0.521 |
| Bilirubin | µmol/l | 1.3 ± 0.1 | 2.2 ± 1.9 | 0.673 |
| Chloride | mmol/l | 102 ± 6 | 94 ± 4 | 0.343 |
| Creatine | µmol/l | 42 ± 1 | 39 ± 1 | 0.100 |
| Globulin | g/l | 15.8 ± 1.4 | 14.9 ± 1.0 | 0.633 |
| Inorganic phosphate | mmol/l | 3.41 ± 0.01 | 3.64 ± 0.43 | 0.615 |
| Potassium | mmol/l | 7.4 ± 1.1 | 8.2 ± 0.8 | 0.592 |
| Protein | g/l | 42.0 ± 2.9 | 40.3 ± 1.8 | 0.650 |
| Urea | mmol/l | 8.0 ± 0.8 | 7.3 ± 0.4 | 0.471 |

Data shown at mean ± SEM. $n = 3$ for each treatment group. Groups compared using an unpaired, two-tailed Student's *t*-test
